# Palmitoylation of A-kinase anchoring protein 79/150 modulates its nanoscale organization, trafficking, and mobility in postsynaptic spines

**DOI:** 10.3389/fnsyn.2022.1004154

**Published:** 2022-09-15

**Authors:** Xiaobing Chen, Kevin C. Crosby, Austin Feng, Alicia M. Purkey, Maria A. Aronova, Christine A. Winters, Virginia T. Crocker, Richard D. Leapman, Thomas S. Reese, Mark L. Dell’Acqua

**Affiliations:** ^1^Laboratory of Neurobiology, National Institute of Neurological Diseases and Stroke (NINDS), National Institutes of Health (NIH), Bethesda, MD, United States; ^2^Department of Pharmacology, University of Colorado School of Medicine, Anschutz Medical Campus, Aurora, CO, United States; ^3^Laboratory of Cellular Imaging and Macromolecular Biophysics, National Institute of Biomedical Imaging and Bioengineering (NIBIB), National Institutes of Health (NIH), Bethesda, MD, United States

**Keywords:** AKAP79/150, palmitoylation, conformation, dendritic spine, endosomes, single-molecule localization microscopy, immunogold EM, STEM tomography

## Abstract

A-kinase anchoring protein 79-human/150-rodent (AKAP79/150) organizes signaling proteins to control synaptic plasticity. AKAP79/150 associates with the plasma membrane and endosomes through its N-terminal domain that contains three polybasic regions and two Cys residues that are reversibly palmitoylated. Mutations abolishing palmitoylation (AKAP79/150 CS) reduce its endosomal localization and association with the postsynaptic density (PSD). Here we combined advanced light and electron microscopy (EM) to characterize the effects of AKAP79/150 palmitoylation on its postsynaptic nanoscale organization, trafficking, and mobility in hippocampal neurons. Immunogold EM revealed prominent extrasynaptic membrane AKAP150 labeling with less labeling at the PSD. The label was at greater distances from the spine membrane for AKAP150 CS than WT in the PSD but not in extra-synaptic locations. Immunogold EM of GFP-tagged AKAP79 WT showed that AKAP79 adopts a vertical, extended conformation at the PSD with its N-terminus at the membrane, in contrast to extrasynaptic locations where it adopts a compact or open configurations of its N- and C-termini with parallel orientation to the membrane. In contrast, GFP-tagged AKAP79 CS was displaced from the PSD coincident with disruption of its vertical orientation, while proximity and orientation with respect to the extra-synaptic membrane was less impacted. Single-molecule localization microscopy (SMLM) revealed a heterogeneous distribution of AKAP150 with distinct high-density, nano-scale regions (HDRs) overlapping the PSD but more prominently located in the extrasynaptic membrane for WT and the CS mutant. Thick section scanning transmission electron microscopy (STEM) tomography revealed AKAP150 immunogold clusters similar in size to HDRs seen by SMLM and more AKAP150 labeled endosomes in spines for WT than for CS, consistent with the requirement for AKAP palmitoylation in endosomal trafficking. Hidden Markov modeling of single molecule tracking data revealed a bound/immobile fraction and two mobile fractions for AKAP79 in spines, with the CS mutant having shorter dwell times and faster transition rates between states than WT, suggesting that palmitoylation stabilizes individual AKAP molecules in various spine subpopulations. These data demonstrate that palmitoylation fine tunes the nanoscale localization, mobility, and trafficking of AKAP79/150 in dendritic spines, which might have profound effects on its regulation of synaptic plasticity.

## Introduction

A-kinase anchoring protein79-human/150-rodent (AKAP79/150) belongs to a class of proteins distinguished by their function in scaffolding protein kinase A (PKA) into distinct signaling domains (Wong and Scott, 2004). In addition to binding PKA, AKAP79/150 also serves to recruit several other enzymes, such as protein kinase C and, notably, the protein phosphatase 2B/Calcineurin (CaN), which often acts in opposition to PKA in the regulation of common signaling targets, including AMPA-type glutamate receptors (AMPAR, reviewed in Purkey and Dell’Acqua, 2020). Neuronal AKAP79/150 anchoring of PKA and CaN has been shown to play a critical role in postsynaptic kinase/phosphatase signaling networks that control both long-term potentiation (LTP) and long-term depression (LTD) of excitatory synaptic strength (Lu et al., 2007, 2008; Sanderson et al., 2012, 2016, 2021). Most excitatory synapses are located on dendritic spines, 1 μm or less in diameter, with synaptic receptors tethered to scaffold proteins in the postsynaptic density (PSD). LTP and LTD increase and decrease synaptic strength by controlling AMPAR recruitment and removal, respectively, from the PSD through regulated trafficking events that involve AMPAR phosphorylation and dephosphorylation, exocytosis and endocytosis, as well as lateral exchange between the PSD and the nearby extrasynaptic membrane (Opazo et al., 2012; Nicoll, 2017; Penn et al., 2017; Purkey and Dell’Acqua, 2020). It is believed that AKAP79/150 plays critical roles in PKA-regulated AMPAR exocytosis from recycling endosomes (REs) in LTP and CaN regulated AMPAR endocytosis into early endosomes (EEs) in LTD, which both occur locally at the extra-synaptic membrane in spines (Kennedy and Ehlers, 2011; Purkey and Dell’Acqua, 2020).

The targeting of AKAP79/150 to the postsynaptic plasma membrane is largely controlled by an N-terminal domain that mediates association with several components, including the acidic phospholipid PI-4,5-P_2_, the cortical F-actin network, and the synaptic cell adhesion molecule N-cadherin (Dell’Acqua et al., 1998; Gomez et al., 2002; Gorski et al., 2005). Additionally, AKAP79/150 is reversibly palmitoylated at two Cys residues in this N-terminal domain, resulting in its segregation into membrane lipid rafts (Delint-Ramirez et al., 2011; Keith et al., 2012).

AKAP palmitoylation is regulated by neuronal activity, with LTP leading to increased palmitoylation and occupancy of the postsynaptic spine compartment, while LTD induces the opposite effects (Keith et al., 2012; Woolfrey et al., 2018). While palmitoylation is not absolutely required for the localization of AKAP79/150 to the plasma membrane in general, mutations blocking palmitoylation (AKAP79/150 CS) reduced AKAP localization to EEs and REs and reduced association with the core PSD, a lipid-raft enriched membrane specialization (Keith et al., 2012; Woolfrey et al., 2015; Purkey et al., 2018). Furthermore, a palmitoylation-deficient AKAP79 CS mutant, which is reduced in REs but still localized to the plasma membrane, disrupted RE exocytosis required for GluA1 AMPAR delivery and dendritic spine enlargement associated with LTP in neuronal cultures (Keith et al., 2012; Woolfrey et al., 2015). Functional studies in a palmitoylation-null AKAP150 CS knock-in mouse further found that palmitoylation also serves to limit the basal incorporation of GluA1 homomeric Ca^2+^-permeable AMPA receptors (CP-AMPARs) (Purkey et al., 2018). Importantly, preventing AKAP palmitoylation in AKAP150 CS mice resulted in the disruption of CP-AMPAR dependent LTP, while LTD and CP-AMPAR independent LTP were unaffected (Purkey et al., 2018).

Within the submicron compartment of the dendritic spine, the PSD is generally < 500 nm in size and is further organized into discrete receptor and scaffold protein-enriched nano-domains that are ∼70–200 nm in diameter. These can be visualized using super-resolution light microscopy but not conventional fluorescence microscopy due to the ∼250–350 nm diffraction-limited resolution of conventional fluorescence microscopy (Fukata et al., 2013; MacGillavry et al., 2013; Nair et al., 2013; Broadhead et al., 2016; Tang et al., 2016; Hruska et al., 2018, but see Masch et al., 2018; Wegner et al., 2018). Accordingly, the nano-organization of AMPARs and scaffolds in the PSD must somehow be regulated to orchestrate the precise signaling events that control AMPAR trafficking in and out of the PSD. Yet, despite great interest in understanding postsynaptic nano-compartmentalization of AMPAR trafficking, scaffolding, and signaling, until the recent advent of super-resolution microscopy, it has been difficult to clearly delineate distinct synaptic, extrasynaptic, and endosomal pools of key regulatory signaling proteins in spines like AKAP79/150. Electron microscopy (EM), while more technically demanding than light microscopy, also excels in visualizing organization of proteins and subcellular organelles in spines and synapses (Harris and Stevens, 1989; Petralia and Wenthold, 1999; Valtschanoff and Weinberg, 2001; Chen et al., 2008a,^2011^, ^2015^; Tao-Cheng et al., 2021). Thus, here we employed a combination of super-resolution light microscopy (i.e., direct stochastic optical-reconstruction microscopy; *d*STORM and photo-activation localization microscopy; PALM) and EM [i.e., immunogold EM and thick section scanning transmission EM (STEM) tomography; Hohmann-Marriott et al., 2009; Chen et al., 2015] imaging approaches to characterize the role of palmitoylation in controlling the nanoscale organization, trafficking and mobility dynamics of AKAP79/150 in dendritic spines that could be crucial for regulation of AMPARs during synaptic plasticity.

## Results

### Nanoscale distribution of AKAP79/150 at excitatory synapses imaged by immunogold electron microscopy and super-resolution light microscopy

**FIGURE 1:**
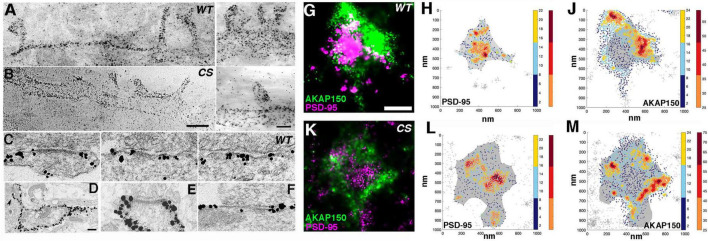
Nano-scale distribution of AKAP79/150 in hippocampal neurons. (**A**, left and **B**, left) Low magnification bright field scanning transmission electron microscopy (STEM) images of ∼1μm thick Epon embedded plastic sections of immunogold labeled endogenous AKAP150 in dissociated hippocampal mouse cultures with a home-made primary antibody showing extensive AKAP labeling at the dendritic and spine membrane. **(A)** Wild type mouse (WT). **(B)** Palmitoylation deficient knock-in mutant mouse (CS). Scale bar 600 nm. (A-right and B-right) Other examples of higher magnification STEM images of immunogold labeled endogenous AKAP150 in the membrane of spines and dendrites. A-right = WT, B-right = CS, Same samples as in (**A**, left) and (**B**, left), respectively. Scale bar 400 nm. Non-specific gold particles are 10 nm gold particles applied as fiducial makers for aiding tomography reconstructions. **(C)** Thin section transmission EM images of immunogold labeled endogenous AKAP150 in excitatory synapses in WT hippocampal mouse neurons. Scale bar 100 nm. **(D–F)** Thin section EM images of overexpressed AKAP79-GFP immunogold labeled for GFP in rat hippocampal neurons. **(D)** Low magnification EM micrograph shows extensive AKAP79-GFP immunogold labels in dendritic and extrasynaptic membrane. Arrowhead points to a spine synapse. Scale bar 300 nm. **(E)** AKAP79 labels stopped at the outer edge of the PSD at a spine synapse. **(F)** AKAP79 labels at a shaft synapse. Same scale as **(C)**. **(G,K)** *d*STORM images of endogenously labeled AKAP150 (CF568) and PSD95 (AF647). Rendered images of spines from a wild-type mouse **(G)** and palmitoylation-null AKAP150 CS knock-in mouse **(K)** *d*STORM AKAP150 localization shown in green and PSD-95 localization shown in magenta. Scale bar 250 nm. Scatterplot heatmaps of localizations of PSD-95 **(H,L)** or AKAP150 **(J,M)**. Wild-type data shown in **(H,J)** and CS data shown in **(L,M)**. The density-based PSD delineation is shown in gray as a reference in all scatter-maps. Color-scale represents the local density of each coordinate (*see section “Materials and methods”*). Localizations delineated as part of a high-density region (HDR) shown in hot colors (right color-bar). The AKAP150 HDRs are often clustered prominently at the periphery of the postsynaptic density.

To study endogenous distribution of AKAP150 at synapses in dissociated mouse neuronal cultures, we used a home-made primary antibody raised against a rodent specific repeat in the mid-region between aa 315–615 of AKAP150 (Brandao et al., 2012). Previous imaging with super-resolution stimulated-excitation depletion (STED) microscopy showed extensive AKAP150 labeling in the dendritic plasma membrane including the extrasynaptic membrane and at synaptic sites overlapping PSD-95 in spines, indicating that at least some AKAP molecules are localized in the core PSD of excitatory synapses as defined by PSD-95 (Purkey et al., 2018). High-definition immunogold labeling of endogenous AKAP150 using this same antibody in hippocampal cultures from WT and Cys to Ser (CS) knock-in mutant mice, where palmitoylation of AKAP150 is abolished, were imaged by STEM in ∼1 μm thick, plastic-embedded sections. Similar to previous STED imaging, STEM imaging showed extensive immunogold particle labeling along the membrane in dendrites and spines, demonstrating directly that AKAP150 is distributed with relatively high density in cell membranes of dendrites and spines alike (Figures 1A,B). From thick section STEM tomography reconstructions, detailed measurements of the nearest neighbor distance of immunogold particles were made to compare the relative enrichment and distribution of AKAP150 WT and CS in different locations. Nearest neighbor distances of AKAP150 label in various compartments of spines and dendrites overall appeared to be not affected by palmitoylation: PSD membrane 63 ± 23 nm (32–107 nm, *N* = 17, WT), 72 ± 35 nm (33–177 nm, *N* = 28, CS, NS, *p* > 0.9999, ANOVA, Kruskal-Wallis test); Extrasynaptic spine membrane 86 ± 35 nm (34–238 nm, *N* = 119, WT), 77 ± 24 nm (42–127 nm, *N* = 18, CS, *p* > 0.9999); Spine cytoplasm 81 ± 30 nm (28–205 nm, *N* = 238, WT), 86 ± 27 nm (37–161 nm, *N* = 65, CS, *p* > 0.9999); Dendritic membrane 95 ± 34 nm (30–175 nm, *N* = 167, WT), 85 ± 32 nm (33–177 nm, *N* = 170, CS, *p* = 0.094); Dendrite cytoplasm 91 ± 34 nm (28–214 nm, *N* = 240, WT), 99 ± 34 nm (31–214 nm, *N* = 85, CS, *p* > 0.9999). Of interest is that the AKAP150 label nearest neighbor distance was not significantly different at the PSD and at extrasynaptic spine membrane (WT, *p* = 0.3712; CS, *p* > 0.9999). Furthermore, the closet nearest neighbor distance observed was ∼ 30 nm, which closely matched the average nearest neighbor distance among AMPAR or NMDAR-type structures in the PSD previously revealed by EM tomography (Chen et al., 2008a,^2011^, ^2015^).

**FIGURE 2:**
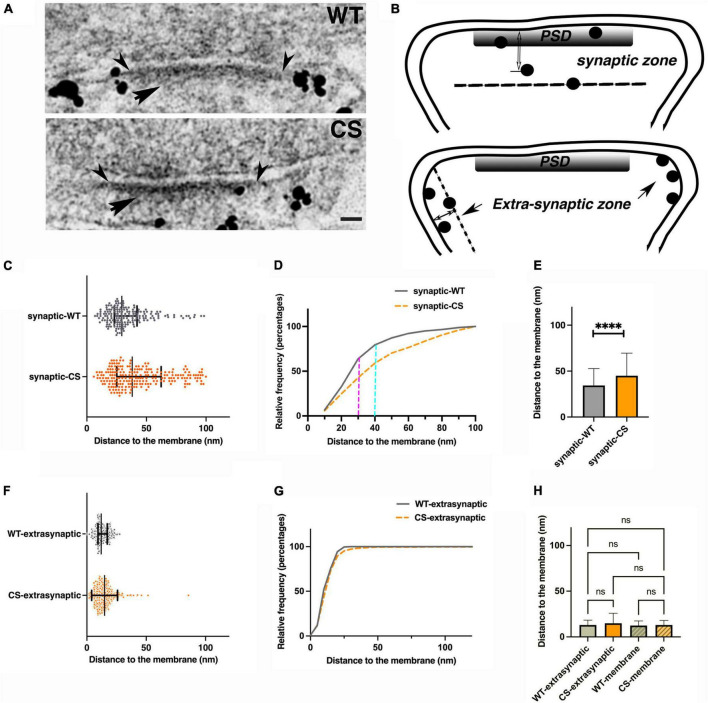
Palmitoylation effects on nano-scale localization of endogenous AKAP150 in hippocampal mouse spine synapses by immunogold EM. **(A)** Electron micrographs of individual synapses labeled for AKAP150 (WT and CS). Arrowheads point to outer edge of the electron dense PSD at the postsynaptic membrane. Arrow points to the PSD. Scale bar 100 nm. **(B)** Schematic drawings of synaptic vs. extra-synaptic zones designated for measuring AKAP150 immunogold particle distances to postsynaptic membrane (PSM). **(C,F)** Immunogold to membrane distance distribution for WT (blue dots) vs. CS (magenta) with mean and SD in the synaptic zone **(C)** and extra synaptic zone **(F)**. **(D)** Cumulative distribution of data in **(C)** showing more spread immunogold distribution for CS than WT (WT: *N* = 180, mean ± SD = 34 ± 18 nm; median = 29 nm, 25% percentile = 23 nm, 75% percentile = 42 nm; CS: *N* = 222, mean ± SD = 45 ± 25 nm; median = 38 nm, 25% percentile = 25 nm, 75% percentile = 62 nm), and significant percentages of labels are within the core PSD structures up to 30–40 nm from PSM: up to 30 nm to PSM (magenta dash line): ∼35% (CS), ∼60% (WT); up to 40 nm to PSM (cyan dash line): ∼50% (CS), ∼70% (WT). **(E)** Histogram comparing mean gold particle distance to PSM for WT and CS (*P* < 0.0001. Mann-Whitney test). **(G)** Cumulative distribution of data in **(F)** showing essentially identical immunogold distribution for WT and CS at the extra-synaptic membrane. **(H)** Histogram comparing immunogold particle to membrane distances at extra-synaptic membrane vs. non-synaptic dendritic membrane show no significant differences. 13 ± 5 nm (WT, extrasynaptic membrane, CV = 0.38, *N* = 309), 12 ± 5 nm (WT, non-synaptic membrane, CV = 0.42, *N* = 229, 7 STEM tomograms); 15 ± 11 nm (CS, extra-synaptic membrane, CV = 0.73, *N* = 403), 13 ± 5 nm (non-synaptic membrane, CS, CV = 0.38, *N* = 164, 10 STEM tomograms). Ordinary one way ANOVA, Tukey test, WT-extra-synaptic (*N* = 309) and CS-extra-synaptic (*N* = 403), *P* = 0.9995; WT-extra-synaptic and WT-membrane (*N* = 229) *P* > 0.9999;WT-extrasynaptic and CS-membrane (*N* = 164), *P* > 0.9999; CS-extrasynaptic and CS-membrane, *P* = 0.9999; WT-membrane and CS-membrane, *P* = 0.9978). ****p < 0.0001, *^ns^p* > 0.05.

From multiple thin-section EM images of synapses, immunogold labeling of endogenous AKAP150 appeared to be extensive at the extrasynaptic membrane of excitatory synapses containing prominent electron dense PSDs in both spines and dendrites shafts (Figure 1C). In some synapses immunogold particle labels were found overlapping the electron dense core PSD structure, but in the majority of cases, extrasynaptic AKAP immunogold labeling was most prominent at the peri-synaptic outer rim of the PSD (Figures 1C–F, 2A). At the extrasynaptic membrane, AKAP labels appeared to be located right at the membrane; while at the PSD, they often were located 10s of nanometers further away from the postsynaptic membrane underneath the core electron dense layer of the PSD (Figures 1C,F). When GFP-tagged AKAP79 constructs were overexpressed in rat hippocampal neuron cultures localized by immunogold EM, the overexpressed AKAP79 showed a similar labeling pattern to that of endogenous AKAP150, namely extensive extrasynaptic labeling near the outer edge of the PSD but with less labeling directly overlapping the core PSD itself (Figures 1D–F).

Using the single-molecule localization microscopy (SMLM) method commonly known as *d*STORM to image neurons from WT or AKAP150 CS mice labeled with antibodies for PSD-95 (Alexa-647) and AKAP150 (CF 568), we also observed a highly heterogeneous distribution of the AKAP in spines, characterized by distinct high-density regions (HDRs, Figures 1G–M). In both CS and WT neurons, the bulk of AKAP localizations and HDRs were found near the periphery of the PSD, which was identified by the high-density of PSD-95 localizations, although scattered AKAP localizations could be observed across the synapse. AKAP150 HDRs localized around the periphery of the PSD typically at the edge of the synaptic/peri-synaptic area (Figure 1G), which was very similar to the prominent peri-synaptic localization of AKAP150 observed above by immunogold EM. In corroboration with immunogold EM, SMLM revealed that the relative distributions of AKAP150 WT and the CS mutant between the extrasynaptic membrane and PSD were similar.

### AKAP150 immunogold clusters revealed by scanning transmission electron microscopy tomography

**FIGURE 3:**
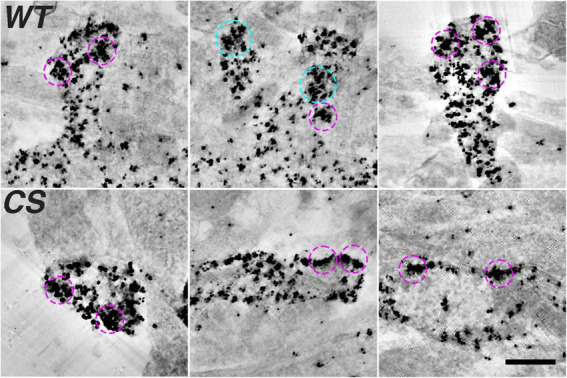
Densely labeled AKAP150 gold clusters at spine synapses revealed by thick section STEM tomography (Top row, WT; Bottom row, CS). Projection views of 50 averaged consecutive virtual sections (222 nm) of AKAP150 labeled dendritic spines showing ∼200 nm dense gold clusters enclosed by magenta circles (inner diameter 240 nm) and ∼300 nm dense gold clusters enclosed by cyan circles (inner diameter 336 nm). Many dense gold clusters are located next to extra synaptic membrane, some clusters were at the PSD, some in the cytoplasm. Scale bar 500 nm.

The observation of high density AKAP150 nanoclusters at the spine membrane by super-resolution light microscopy prompted us to further examine the distribution of immunogold label of endogenous AKAP150 WT and CS in thick section STEM tomograms. We generated projection images from STEM tomograms by averaging 50 consecutive virtual sections (222 nm thick) and found various examples of dense gold particle clusters located in dendritic spines for both AKAP150 WT and CS (Figure 3). The sizes of these gold clusters ranged from 100 to 300 nm, which was in general agreement with the size of high density AKAP150 nanoclusters observed by SMLM (Figures 1G–M). These AKAP150 gold clusters were often located close to the extrasynaptic membrane, some in the PSD, and some in the cytoplasm of dendritic spines, thus confirming that AKAP150 is organized into dense nano-scale clusters in multiple neuronal compartments. Although the molecular underpinnings remain to be characterized, cluster formation could be mediated by AKAP dimerization in conjunction with additional molecular assemblies that together would serve to anchor and concentrate the AKAP and associated signaling partners (Gold et al., 2011; Zhang et al., 2016).

### Palmitoylation differentially affects endogenous AKAP150 localization at the postsynaptic density and extrasynaptic spine membrane

To further probe how palmitoylation controls AKAP nano-scale organization, we used immunogold and thin-section EM (Figure 2A) to measure immunogold particle to membrane distances either in the synaptic/PSD or extrasynaptic zones in neurons cultured from WT and CS knock-in mice (Figure 2B). The synaptic zone was defined as a region extending from inner leaflet of the postsynaptic membrane associated with the PSD to 100 nm deep in cytoplasm (Figure 2B). Likewise, the extrasynaptic zone was defined as any region outside the PSD within a vertical distance of 100 nm from the spine plasma membrane. At the synaptic zone, the CS mutant exhibited significantly larger average distances between the immunogold particles and the postsynaptic membrane, with a much wider spatial distribution and a larger standard deviation compared to WT (Figures 2C–E): 34 ± 18 nm (WT, coefficient of variation-CV = 0.53, number of gold particles-N = 180, number of synapses = 175) vs. 45 ± 25 nm (CS mutant, CV = 0.56, *N* = 222, number of synapses = 164, ^****^p < 0.0001, Mann-Whitney test) consistent with greater spread in the distribution for AKAP150 CS due to its displacement away from the postsynaptic membrane in the PSD. In sharp contrast with the synaptic zone, the gold particle to plasma membrane distances in the extrasynaptic zone were much more similar for WT and CS (Figures 2F–H): 13 ± 5 nm (WT, CV = 0.38, *N* = 309) and 15 ± 11 nm (CS, CV = 0.73, *N* = 403, ANOVA, Kruskal Wallis test, *p* = 0.2994) with no significant difference in AKAP WT and CS localization at the extrasynaptic membrane. These EM data confirm previous studies showing that palmitoylation is not required for AKAP79/150 targeting to the plasma membrane in general and are consistent with the idea that the N-terminal polybasic domains can directly interact with membrane phospholipids independent of palmitoylation (Dell’Acqua et al., 1998; Keith et al., 2012). Although loss of palmitoylation did not significantly alter AKAP localization to the extrasynaptic membrane, the larger CV in the CS mutant compared to WT is perhaps still indicative of a somewhat broader distribution relative to the plasma membrane, suggesting that palmitoylation may still play a secondary role in AKAP stabilization at the extrasynaptic membrane.

We also compared the average immunogold particle to the membrane distances at the extrasynaptic spine membrane vs. at non-synaptic dendritic shaft membrane in WT and CS measured from thin section EM images and from thick section STEM tomography reconstructions and found none of the average distances were significantly different from each other (Figure 2H). These data showed that both in WT and CS, AKAP localization at the extrasynaptic membrane of dendritic spines is not significantly different from that at the non-synaptic membrane in dendrite shafts. Finally, we counted the number of endogenous AKAP150 immunogold labels from thin section EM images at synaptic or extrasynaptic zones in mouse hippocampal synapses as a means to estimate relative AKAP150 abundance in these two zones: WT, 180 (synaptic), 309 (extrasynaptic); CS, 222 (synaptic), 403 (extra-synaptic), thus synaptic labels accounts for ∼37% (37%, WT; 36% CS) and extrasynaptic labels accounts for ∼63% (63%, WT; 64%, CS) of total labels. Therefore, in agreement with other analyses above, the majority of AKAP150 labels for both WT and CS are present in the extrasynaptic membrane rather than at the PSD in dendritic spines. In addition, an albeit reduced but still sizeable portion of immunogold labels in the CS mutant remained within 30–40 nm from the postsynaptic membrane in the PSD, which is regarded as the core layer of the PSD (Chen et al., 2011; Figure 2D): < 30 nm ∼35% (CS), ∼60% (WT); < 40 nm ∼50% (CS), ∼70% (WT), indicating that there was still a discernable population of AKAP150 in the PSD for the CS mutant. Therefore, while palmitoylation is clearly one of the important determinants retaining AKAP150 in the PSD, interactions other than palmitoylation also must contribute.

### Distinct AKAP79 conformations in the postsynaptic density vs. extrasynaptic membrane

**FIGURE 4:**
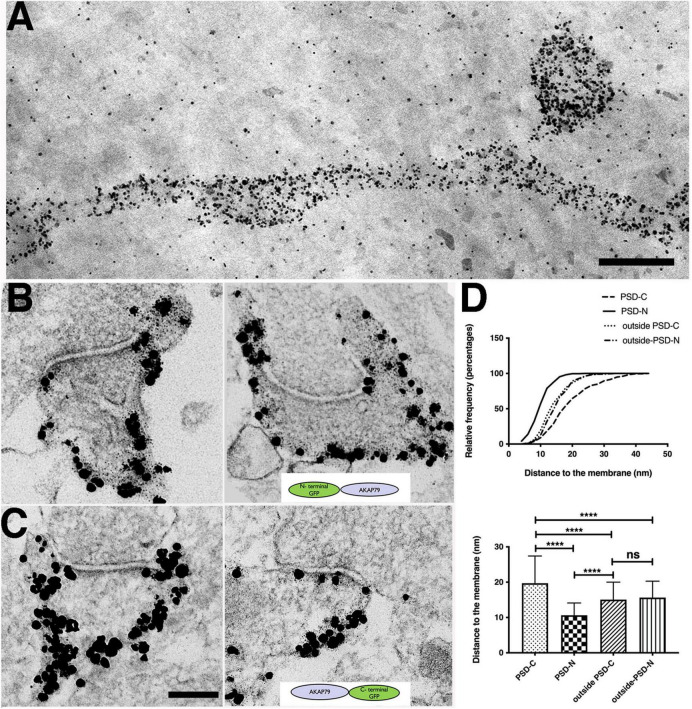
Molecular configuration of AKAP79 at spine synapses determined by immunogold EM. **(A)** Thick section STEM image of immunogold labels of overexpressed AKAP79-GFP (with anti-GFP antibody) showing extensive AKAP79 labels at the dendritic and spine membrane. Scale bar 400 nm. Non-specific gold particles are added as 10 nm fiducial markers for aiding tomography reconstructions. **(B)** Immunogold labeling of GFP in N-terminally tagged GFP-AKAP79 at synapses **(C)** Immunogold labeling of GFP in C-terminally tagged AKAP-GFP. Scale bar 200 nm. **(D)** Cumulative distribution (top) and histogram (bottom) comparing localization of N-terminally tagged GFP-AKAP79 in the PSD (PSD-N) and outside the PSD at the extra-synaptic membrane (outside-PSD-N) vs. C-terminally tagged AKAP79-GFP in the PSD (PSD-C) and outside the PSD at the extra-synaptic membrane (outside-PSD-C). ****p < 0.0001, *^ns^p* > 0.05.

Interestingly, we noticed above that the average distance of AKAP150 WT label to the postsynaptic membrane in the PSD/synaptic zone of 34 ± 18 nm (Figure 2C) was significantly deeper into the cytoplasm than the distance of 13 ± 5 nm measured at the extrasynaptic zone (Figure 2F, one way ANOVA, Tukey test, ^****^p < 0.0001), raising the possibility that the AKAP might adopt different conformations in the PSD vs. at the extrasynaptic membrane. However, as mentioned above the AKAP150 antibody recognizes an internal repeat region that is separated from the N-terminal membrane targeting domain by 150–300 aa, thus this antibody cannot actually inform on where the AKAP N-terminal targeting is with respect to the plasma membrane in the synaptic zone/PSD vs. extrasynaptic zone. To address this question further, we made additional comparisons of the anti-GFP immunogold particle distances for N vs. C GFP-tagged AKAP79 WT constructs transiently expressed in rat hippocampal neuron cultures and found that within the synaptic zone the N-terminus was on average 11 ± 3 nm (*N* = 275) from the postsynaptic membrane while the C -terminus was nearly twice as far away at 20 ± 8 nm from the postsynaptic membrane (*N* = 212, ^****^p < 0.0001, one-way ANOVA, Tukey test, Figures 4A–D). These results suggest that, at the PSD, AKAP79 adopts an open configuration with its N-terminus at the membrane and its C-terminus oriented away from the membrane in a vertical orientation to the postsynaptic membrane similar to that of PSD-95 (Chen et al., 2008a,^2011^, ^2015^). In contrast, at the extrasynaptic membrane, the relative locations of both N and C termini to the membrane were essentially identical (Figure 4D): 16 ± 5 nm (*N* = 301, N-GFP) and 15 ± 5 nm (*N* = 175, C-GFP, *P* = 0.6451, NS), and both were found at somewhat greater distances from the membrane than the AKAP79 N-terminus within the PSD (^****^p < 0.0001). Overall, in the PSD the AKAP79 N-terminus is located closest to the membrane and the C-terminus is located at the greatest distance from the membrane, while in the extrasynaptic membrane the AKAP79 N- and C-termini are both located at similar distances from the membrane that are slightly greater than the AKAP N-terminus membrane distance in the PSD. These results suggest that at the extrasynaptic membrane AKAP79 either adopts a folded conformation where its N and C termini are in close proximity or an extended, open configuration oriented parallel to the membrane. Regardless, these results demonstrate that AKAP79/150 adopts distinct molecular conformations at the PSD vs. at the extrasynaptic membrane, indicating that different molecular interactions are likely contributing to AKAP retention in these two locations.

### Palmitoylation differentially affects AKAP79 localization to the postsynaptic density and extrasynaptic membrane

**FIGURE 5:**
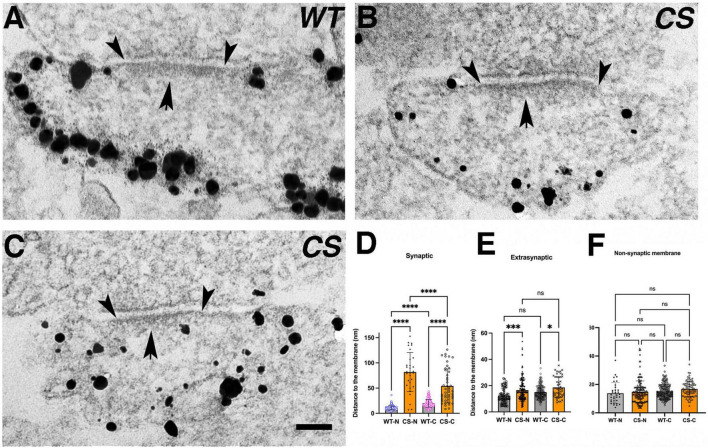
Palmitoylation effects on nano-scale localization and molecular configuration of AKAP79 in rat hippocampal spine synapses determined by immunogold EM. **(A)** Electron micrograph of immunogold labeled overexpressed WT AKAP79-GFP (C-terminally GFP tagged) at a spine synapse. Arrowheads delineate the outer edges of the PSD, arrow points to the PSD. **(B,C)** Electron microgram of immunogold labeled overexpressed CS AKAP79-GFP at a spine synapse. Scale bar 100 nm. **(D–F)** Histograms comparing immunogold to the membrane distance between WT and CS in synaptic, extrasynaptic zones, and non-synaptic membrane for N-terminally GFP tagged AKAP79 (WT-N, CS-N) or C-terminally GFP tagged AKAP79 (WT-C, CS-C). ANOVA, Tukey test: 11 ± 4 nm (range 3.5–36 nm, CV = 0.36, *N* = 280, GFP-N, WT), 82 ± 39 nm (range 7–153 nm, CV = 0.48, *N* = 30, GFP-N, CS, ****p < 0.0001); 20 ± 8 nm (range 5–43 nm, CV = 0.4, *N* = 424, C-GFP, WT), 54 ± 34 nm (range 9–126 nm, CV = 0.63, *N* = 46, C-GFP, CS, ****p < 0.0001). 12 ± 6 nm (range 4–25 nm, *N* = 68, CV = 0.5, GFP-N, WT), 17 ± 8 nm (range 4–53 nm, *N* = 180, CV = 0.47, GFP-N, CS, ***p = 0.0004); 15 ± 5 nm (range 5–33 nm, *N* = 175, CV = 0.33, C-GFP, WT), 19 ± 8 nm (range 7–35 nm, *N* = 53, CV = 0.42, C-GFP, CS, *p = 0.013). 14 ± 7 nm (range 4–37 nm, *N* = 33, CV = 0.5, GFP-N, WT), 15 ± 8 nm (range 5–33 nm, *N* = 155, CV = 0.53, GFP-N, CS, *P* = 0.9962); 15 ± 5 nm (range 5–33 nm, *N* = 175, CV = 0.33, C-GFP, WT), 17 ± 5 nm (range 5–34 nm, *N* = 83, CV = 0.29, C-GFP, CS, *P* = 0.4818). *p ≤ 0.05, ***p < 0.001, ****p < 0.0001, *^ns^p* > 0.05.

As mentioned above, the antibody we used to label endogenous AKAP150 targets an internal rodent-specific repeat region, which is separated by 150–300 aa from the palmitoylated N-terminal targeting domain that associates with membranes (Figures 1, 2). Thus, we wondered if by using this antibody to localize AKAP150 with immunogold EM we could be inadvertently masking or underestimating some of the impacts of palmitoylation on AKAP nano-scale localization. We therefore transiently expressed N or C terminally GFP-tagged AKAP79 CS constructs in rat hippocampal neurons and compared localization by immunogold EM to corresponding GFP-tagged AKAP79 WT constructs (Figures 5A–C). In agreement with the endogenous AKAP150 CS labeling data above (Figure 2), we found in the synaptic zone the average distances of the immunogold labels detecting both N and C-terminally GFP-tagged AKAP79 were significantly further away from the membrane for CS compared to WT (Figures 5B,D), further confirming that palmitoylation is an important determinant in localizing AKAP79/150 within the PSD. At the extrasynaptic membrane, the average distances of the immunogold label of N and C terminally tagged GFP were also significantly further away from the membrane in CS than in WT (Figures 5C,E), but these distances to extrasynaptic membrane for CS and WT were still much more similar to each other than those measured in the synaptic zone. Finally, there was no significant differences in localization at non-synaptic dendritic shaft membranes for AKAP79 WT and CS (Figure 5F). Thus, these data for GFP-tagged AKAP79 WT and CS overall reinforce our conclusions above for endogenous AKAP150 WT and CS (Figure 2), that palmitoylation likely plays only a secondary role in stabilizing AKAP in the extrasynaptic and non-synaptic membrane.

However, in the synaptic zone, in contrast to WT, GFP-tagged AKAP79 CS constructs (Figures 5B,D) showed significant displacement from the core PSD, e.g., 82 ± 39 nm (CV = 0.48, GFP-N, CS), 54 ± 34 nm (CV = 0.63, C-GFP, CS) from the synaptic plasma membrane. The very large CV in the data made it not possible to meaningfully determine AKAP79 CS conformation, however, the data certainly indicated that the CS mutation led to loss of the distinct membrane-associated vertical and extended orientation at the PSD observed for WT (Figure 5D). For AKAP79 CS similar distances of the N and C termini to the spine membrane at the extrasynaptic zone suggested either a closed or extended conformation oriented parallel to the membrane that is similar to WT (Figures 5B,C,E), e.g., 17 ± 8 nm (CV = 0.47, GFP-N, CS), 19 ± 8 nm (CV = 0.42, C-GFP, CS).

### Identification of AKAP150 immunogold labeled endosomal structures in spines

**FIGURE 6:**
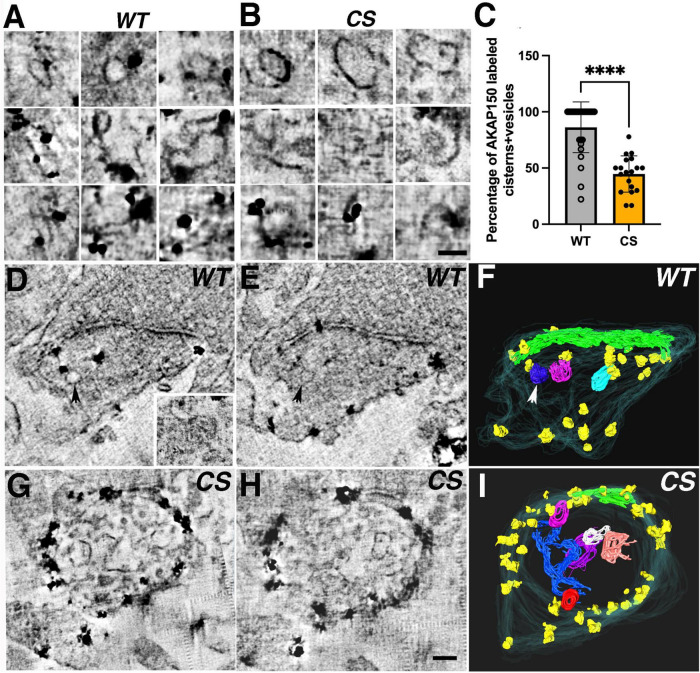
AKAP150 labeled endosomal structures in spine synapses revealed by STEM tomography. **(A)** In WT spines, 3 × 3 image gallery of AKAP150 immunogold labeled endosomal structures (e.g., large vesicle, coated vesicle, amorphous cistern, tubular cistern) from projections of 5–9 averaged virtual sections. **(B)** In CS spines, 3 × 3 image gallery of vesicles, coated vesicles and cisterns not associated with AKAP150 labels but some endosomal structures (vesicles and tubules) are still immunogold labeled for AKAP150. Scale bar 100 nm. **(C)** Percentage of AKAP150 labeled endosomal structures are reduced in CS compared with WT (Mann-Whitney test, ^****^P < 0.0001, WT, 27 spine synapses in 8 STEM tomography reconstructions: CS, 20 spine synapses in 6 STEM tomography reconstructions). **(D–F)** AKAP150 labeled WT spine synapse reconstructed by STEM tomography. **(D)** A virtual section of the AKAP150 labeled spine synapse, arrow points to an immunogold labeled vesicle. Inset: a multi-vesicular body associated with a tubule labeled for AKAP150, a typical endosomal structure. **(E)** A neighboring virtual section close to **(D)**, arrow point to the same labeled vesicle. **(F)** Surface rendering of the AKAP labeled dendritic spine from the tomography reconstruction. spine membrane (translucent cyan), PSD (green), immunogold particles (yellow), AKAP labeled endosomal vesicles (blue, magenta, cyan). Arrow points to the labeled vesicle in **(D,E)**. **(G–I)** AKAP150 labeled CS spine synapse reconstructed by STEM tomography showing many vesicles and cisterns in spine cytoplasm lacking AKAP150 labels. **(I)** Surface rendering of the spine synapse from the STEM tomography reconstruction in **(G,H)**. Spine membrane (translucent cyan), PSD (green), AKAP150 immunogold particles (yellow), unlabeled endosomal vesicles and cisterns (blue, red, white, purple, pink, orange). Scale bar 100 nm.

Conventional thin section (∼ 80 nm) EM images only provide limited information on the sub-cellular organelle contents of dendritic spines. Though serial section EM has enjoyed great success in elucidating sub-cellular organelles in spines (Harris and Stevens, 1989; Harris et al., 1992), the technical limit of ∼ 50 nm in the thickness of sections might still obscure identification of many immunogold labeled sub-cellular structures. Recently developed STEM tomography on 1–2 μm thick sections allows 3D reconstruction of synapses with a 2–4 nm virtual section thickness (Chen et al., 2015), thus providing means to more definitively identify immunogold labeled sub-cellular structures in tomograms. Here we visualized in 3D AKAP150-labeled organelles in dendritic spines of cultured mouse hippocampal neurons. From 27 (WT) and 20 (CS) spine synapses reconstructed using STEM tomography (Figures 6A–I), we found many AKAP150 immunogold labeled vesicular membrane compartments in the cytoplasm of dendritic spines. In WT spine synapses, AKAP150 immunogold labeled vesicles and cisterns, including large vesicles (∼100 nm), coated vesicles, amorphous cisterns, tubular cisterns (Figures 6A,D–F, virtual sections and rendering of 3D-reconstructions) as well as tubules associated with multi-vesicular bodies (MVBs, Figure 6D inset). These type of membrane structures have been identified and characterized as part of EE and RE networks in dendritic spines involved in the cycling of proteins in and out of the postsynaptic membrane (Cooney et al., 2002). In contrast, in CS spine synapses, many of the amorphous vesicular endosomal structures were not labeled, though we could still find some examples of AKAP150 immunogold label associated with large vesicles or tubular endosomes (Figures 6B,G–I, virtual sections and rendering of 3D-reconstructions), which are characteristic of REs (Ullrich et al., 1996). Since AKAP150 CS is not palmitoylated, this data demonstrates that some population of non-palmitoylated AKAP150 can still be associated with REs, although not nearly to the same degree as WT. To make this comparison between WT and CS more quantitative, we calculated the percentage of AKAP150-labeled endosomal structures out of the total number of membrane structures in each dendritic spine, and found significant reduction (∼47%) of AKAP150-labeled endosomal structures in CS compared to WT: 86% ± 23% (WT, *N* = 27), 45 ± 16% (CS, *N* = 20, ^****^p < 0.0001, Mann Whitney test) (Figure 6C), thus providing direct evidence at the ultra-structural level that AKAP150 palmitoylation is a major determinant in regulating RE localization and trafficking (Keith et al., 2012; Woolfrey et al., 2015; Purkey et al., 2018).

### Single-molecule tracking reveals that palmitoylation controls the kinetics of AKAP79 exchange between multiple mobile states and a bound/immobile state in spines

**FIGURE 7:**
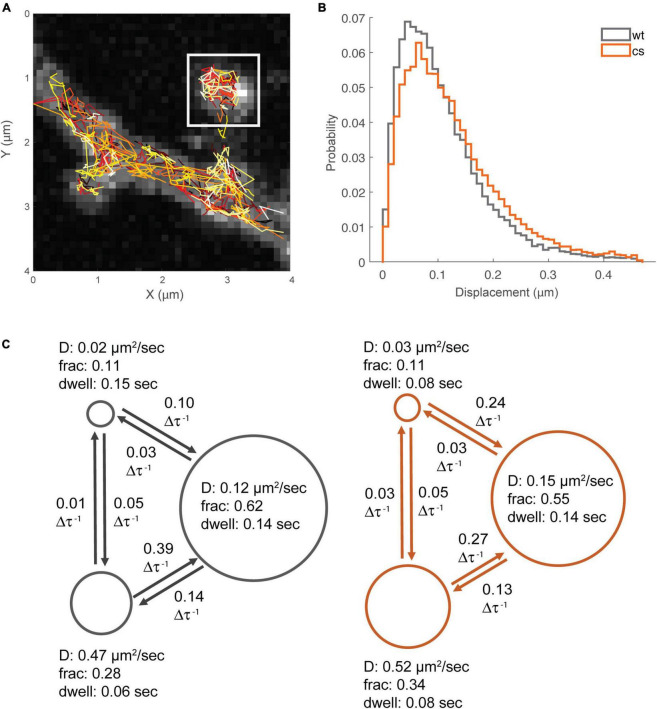
Single-molecule tracking reveals multiple mobility states for AKAP79. **(A)** Example smt-PALM dataset, only trajectories from distinct spine heads were used for downstream analysis (white box). **(B)** Semi-quantitative analysis of single-step displacements. **(C)** Hidden-Markov analysis of trajectories, WT states/transitions are shown in gray, CS states/transitions shown in orange. Circle diameter is proportional to fraction occupancy. Other parameters are apparent diffusion coefficient, average molecular dwell time per state, and inter-state transition probability (per time-step, 22 ms).

Previous work demonstrated that AKAP79/150 localization to dendritic spines is differentially controlled by plasticity-inducing stimuli that also control its palmitoylation state. For example, chemical LTP activation of NMDARs that increases AKAP79/150 palmitoylation also increases AKAP79/150 enrichment at dendritic spines (Keith et al., 2012; Woolfrey et al., 2015). In contrast, chemical LTD activation of NMDARs leads to AKAP79/150 removal from dendritic spines as a result of depalmitoylation and disruption of its binding to F-actin, cadherins, and PI-4,5-P_2_ (Gomez et al., 2002; Gorski et al., 2005; Horne and Dell’Acqua, 2007). Thus far, however, there have been no studies of the nano-scale dynamics of AKAP79/150 mobility within dendritic spine nor how palmitoylation might regulate it. To address this issue, we performed PALM-based single-molecule tracking studies (smtPALM) (Manley et al., 2008) using AKAP79 constructs fused the green-to-red photoconvertible fluorescent protein mEos3.2. To isolate intra-spine dynamics, only trajectories from within distinct spine-heads were analyzed (Figure 7A). A qualitative assessment of the single-step displacements (Figure 7B) of red photo-converted AKAP79WT-mEos3.2 molecules in spines (time-step, Δτ, = 0.022 s) showed that the preponderance of jump-lengths (25–75 percentiles) lay between 0.05 and 0.14 μm (median 0.09 μm). Still, a sizable fraction (12%) showed larger single-step displacements of 0.20 μm and greater. The distribution of jump-lengths for the AKAP79CS-mEos3.2 construct were slightly right-shifted compared to that of the WT construct (Figure 7B), with a lower proportion of short (<0.1 μm) displacements (WT: 56%, CS: 47%), both of which are consistent with greater mobility of the palmitoylation-deficient CS mutant.

Mean squared displacement (MSD) is one of the most frequently used metrics to quantitatively analyze single-molecule trajectory data, however, MSD analysis has been shown to be unreliable when localization precision is poor or when trajectories are short, such as typically seen when for smtPALM using mEos3.2 due to relatively rapid post-conversion photobleaching (Michalet and Berglund, 2012). Furthermore, MSD analysis is not well suited to distinguish between multiple diffusion and binding states (Hansen et al., 2018). A number of more sophisticated analytical methods, more appropriate for the type of data produced by smtPALM (large numbers of short trajectories) have been described (Persson et al., 2013; Hansen et al., 2018). We chose to utilize a Hidden-Markov Modeling based approach, vbSPT, originally described in Persson et al. (2013). This method has the capability of identifying multiple (apparent) diffusion states from smtPALM data having a large number of short-length trajectories. In principle, this technique is able to statistically determine the most likely number of states, however, we limited the analysis to a maximum of 3 states in order to prevent over-fitting. In the case of the WT data, the estimated 3-state system had apparent diffusion coefficients of ≈0.02 μm^2^s^–1^, ≈0.12 μm^2^s^–1^, ≈0.47 μm^2^s^–1^ (Figure 7C), with population fractions of S1: ≈0.11; S2: ≈0.62; S3: ≈0.28. While vbSPT does not return an explicit bound state, it can be inferred that the state with the lowest diffusion coefficient corresponds to a bound fraction. The apparent diffusion coefficient values for the three-states were quite similar for both AKAP79 WT and CS. However, there was a slight shift in the respective state fractions for the CS mutant compared to WT, especially between state2 (WT: D ≈0.12 μm^2^s^–1^, Fr ≈0.62; CS: D ≈0.15 μm^2^s^–1^, Fr ≈0.55) and the highest mobility state3 (WT: D ≈0.47 μm^2^s^–1^, Fr ≈0.28; CS: D ≈0.52 μm^2^s^–1^, Fr: ≈0.34) (Figure 7C). In addition to the apparent diffusion coefficients and fractions, the vbSPT HMM estimation also returns state transitions and dwell times (Figure 7C). The only appreciable differences in these metrics were in the dwell-time of the bound/immobile state (WT: 0.15 s; CS: 0.08 s) and the transition probability from the bound state to the slower mobile state (WT: 0.1 per time-step; CS: 0.24 per time-step). The per time-step transition probability from the faster mobile state to the slower mobile state was also less for the CS protein (WT: 0.39; CS: 0.27). Interestingly the transition probabilities per time-step between the bound state and the faster mobile state was quite low, 0.05 or less, suggesting that AKAP79 needs to transition first from the fast mobile state to the slower mobile state before being captured in the bound/immobile state. Importantly, our data for the CS mutant compared to WT indicate that palmitoylation of AKAP79 increases it rate of transition from the faster to the slower mobile state, slows its rate transition from the bound state to the slower mobile state, and accordingly increases its dwell time in the immobile/bound state, all of which are consistent with our immunogold EM data above indicating that palmitoylation helps stabilize AKAP79 to the spine membrane especially in the PSD but also to a lesser extent at the extrasynaptic membrane.

## Discussion

**FIGURE 8:**
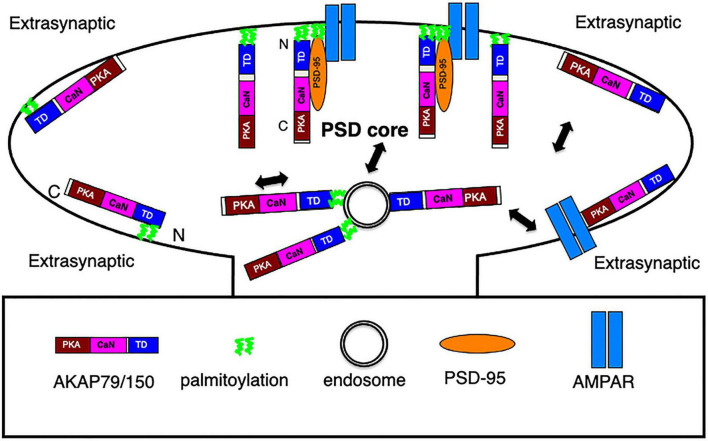
Schematics of AKAP79/150 nano-scale organization, molecular configuration, and trafficking at an excitatory spine synapse. In the PSD, N-terminally palmitoylated AKAP associates with postsynaptic membrane in a vertical and extended configuration, some AKAP molecules are in complex with PSD-95 MAGUK, which also appears as vertical filament and anchors glutamate receptors at the PSD. At the extrasynaptic membrane, AKAP associates with the membrane primarily through N-terminal polybasic regions and secondarily through palmitoylation and is extended but oriented parallel to the membrane. Some may associate with extrasynaptic AMPARs. Both palmitoylated and non-palmitoylated AKAP150 molecules associate with recycling endosomes in spine, but with stronger/more stable association in the palmitoylated state.

AKAP79/150, a membrane associated scaffolding protein, harbors multiple modular domains to not only anchor protein kinases and phosphatases, but also bind to PSD-95 MAGUK, the major scaffolding protein responsible for anchoring glutamate receptors in the PSD (Chen et al., 2008a,^2011^, ^2015^). Thus, AKAP150 is well positioned to target glutamate receptors at the postsynaptic membrane to regulate processes in LTP, LTD and homeostatic plasticity as characterized in previous studies (Lu et al., 2007, 2008; Sanderson et al., 2012, 2016, 2018, 2021). An earlier estimation of the copy number of AKAP79/150 was ∼20 per PSD using quantitative mass spectrometry on isolated Triton-X insoluble PSD fractions (Cheng et al., 2006) suggesting that AKAP150 might not be a significant constituent of the PSD, but a recent estimate using quantitative mass spectrometry of synaptosome preparations from rat brain put this number to be ∼100 (Martinez-Sanchez et al., 2021), much closer to the copy number of PSD-95 in the PSD (Chen et al., 2005). Despite numerous functional studies on AKAP79/150, its molecular organization and dynamics in dendritic spines was still uncharacterized prior to our work here where we combined immunogold EM, thick section STEM tomography, and super-resolution light microscopy to elucidate the effects of AKAP150 palmitoylation on its organization, trafficking, and mobility in spines. Importantly, our multi-modal imaging data shed new light on AKAP79/150 membrane organization, molecular configuration, endosomal trafficking, and mobility dynamics in spine synapses (Figure 8) that provides the basis for further functional studies on AKAP79/150 in the postsynaptic signaling network.

Although previous immuno-EM labeling of AKAP79/150 in hippocampal neurons demonstrated its presence in excitatory synapses, technical limits prevented more precise characterizing of its localization (Sik et al., 2000; Lilly et al., 2005). Prior immunofluorescence microscopy of endogenous AKAP150 in mouse hippocampal neuronal cultures showed its prevalent distribution at the plasma membrane and in REs in the cytoplasm of dendrites and spines (Purkey et al., 2018). Our immunogold EM of endogenous AKAP150 in mouse hippocampal neurons imaged by STEM tomography now showed the extensive high-density presence of AKAP150 label in the dendritic cytoplasm and dendritic and spine plasma membrane, especially at the extrasynaptic membrane for both WT and CS, demonstrating clearly that mechanisms other than palmitoylation is responsible for sustaining AKAP150 at the extrasynaptic spine and dendritic shaft membrane. Further quantitative analysis of AKAP150 organization in neurons for both WT and CS from STEM tomograms showed the average nearest neighbor distances (70–100 nm) of AKAP150 immunogold label at the PSD membrane, extrasynaptic membrane, and spine cytoplasm were not significantly affected by palmitoylation, which is likely a reflection of local AKAP150 interactions with molecular assemblies or organelles in those compartments independent of palmitoylation. Of particular interest is the minimum ∼ 30 nm nearest neighbor distance among AKAP150 labels at the PSD and in extrasynaptic membrane, where AKAP150 could likely be in complex with AMPARs or NMDARs. This ∼30 nm number essentially matches the nearest neighbor distances of NMDAR and AMPAR-type structures in PSDs in rat hippocampal spine synapses revealed by EM tomography (Chen et al., 2008a,^2011^, ^2015^), suggesting that perhaps some of the AKAP150 label at the PSD and/or extrasynaptic membrane represents AKAP150 in complex with glutamate receptors. Further study on this subject is needed.

Endogenous AKAP150 CS immunogold labeling in the synaptic zone at individual spine synapses was more broadly distributed and displaced into the cytoplasm at greater distances from the PSD membrane than WT, demonstrating that palmitoylation is at least partially responsible for retaining some population of AKAP150 at the PSD, which is also consistent with earlier findings that AKAP150 association with isolated PSD fractions measured by immunoblotting and fractional overlap with PSD-95 imaged by STED microscopy were both reduced in CS knock-in mice compared to WT (Purkey et al., 2018). However, our EM analyses also showed that despite the AKAP150 reduction in the PSD in CS, a significant portion of AKAP150 label remains < 30–40 nm from the postsynaptic membrane in the core structural layer of the PSD (Chen et al., 2011). This is a clear indication that interactions other than palmitoylation also help retain at least some sub-population of AKAP150 in the PSD, an obvious candidate being the interaction between AKAP150 and PSD-95 (Colledge et al., 2000; Robertson et al., 2009). In contrast, AKAP150 labeling at the extrasynaptic spine plasma membrane, as well as non-synaptic dendritic shaft membranes, was largely unaffected in CS compared to WT, indicating that palmitoylation of AKAP150 is not the primary molecular means to target and sustain AKAP150 in the extrasynaptic membrane of spines and more generally in the somato-dendritic plasma membrane as a whole. In addition, a greater proportion of AKAP150 immunogold labels visualized by EM and single-molecule localizations imaged by SMLM/*d*STORM were located in the extrasynaptic/peri-synaptic zone than in the synaptic zone/PSD of dendrite spine synapses also likely having implications in further understanding how AKAP150 contributes to postsynaptic signaling networks controlling AMPARs.

We explored the possibility that our home-made AKAP150 antibody targeting an internal repeat region might mask some of the effects of disrupting palmitoylation in AKAP150 CS mice, therefore we also expressed GFP-tagged AKAP79 WT and CS mutant constructs in rat neuron cultures and compared AKAP79-GFP localization to endogenous AKAP150 localization in mouse culture. In both cases, AKAP localization near the synaptic plasma membrane in the PSD was significantly reduced for the CS mutants compared to WT controls, however, at the extrasynaptic membrane there was much less difference in localization between CS and WT, confirming that palmitoylation likely only plays a secondary role in stabilizing and sustaining AKAP79/150 in the extrasynaptic membrane. Mapping the location of N or C terminal GFP-tagged AKAP79 in rat hippocampal synapses by immunogold EM revealed differences in the molecular configuration of AKAP79 in the PSD and at extrasynaptic membrane. At the PSD, the N terminus of AKAP79 is located at the postsynaptic membrane while C terminus is located much further away from the membrane in an extended, open configuration. The proximity of the AKAP79 N-terminus to the plasma membrane is consistent with its N-terminal palmitoylation and poly-basic regions engaging in direct association with the membrane lipid bilayer. The extended C terminus might then be able to bring CaN and PKA near other PSD proteins and complexes, notably glutamate receptor complexes. The AKAP79 molecular conformation at the PSD determined here is essentially identical with that of PSD-95 in the PSD (Chen et al., 2011), therefore AKAP79/150 would appear as a vertical filament in the PSD like PSD-95. Given that AKAP150/79 also associates with PSD-95 (Colledge et al., 2000; Xu et al., 2008; Bhattacharyya et al., 2009; Robertson et al., 2009), it is very likely that some of the vertical filaments in the PSD seen by EM tomography (Chen et al., 2008a,^2011^, ^2015^) could represent a complex containing both PSD-95 and AKAP79/150 (Figure 8). At the extrasynaptic membrane, AKAP79 adopts a very different configuration, likely parallel to the membrane either in a closed or more likely open, extended conformation given prior cryo-EM and FRET analyses demonstrating that the AKAP N- and C- termini are greater than 10 nm apart (Gorski et al., 2005; Nygren et al., 2017). These distinct AKAP79/150 configurations at the PSD vs. at the extrasynaptic membrane are likely also associated with different molecular assemblies in complex with the AKAP at the PSD vs. extrasynaptic membrane (Figure 8), providing an interesting topic for future investigation.

Super-resolution SMLM/*d*STORM imaging of AKAP150 revealed 100–200 nm high density nano-clusters/HDRs, some of which overlapped the PSD but most of which were found in peri-synaptic/extrasynaptic membrane just outside the PSD. When AKAP79/150 was imaged by STORM microscopy, both overexpressed in cells other than neurons and in the cell bodies of sensory neurons, HDRs/nanoclusters were also observed at the plasma membrane (Zhang et al., 2016; Tenner et al., 2020). However, low magnification STEM images of the dense immunogold AKAP150 label in dendritic and spine membranes gave the impression that AKAP150 might be “continuously” distributed along the dendritic and spine membranes. But since thin section immuogold EM is inadequate to resolve this issue, we made use of a subset of projection images from ∼1 μm thick sections reconstructed through bright field STEM tomography and found evidence of 100–300 nm diameter AKAP150 immunogold clusters in dendritic spines, matching closely with the size and distribution of HDRs seen by SMLM and independently confirming the existence of AKAP nanoclusters at the dendritic spine membrane. The functional relevance of these AKAP nanoclusters still needs to be clarified in the future, e.g., it is possible they represent accumulated AKAP150 at the membrane in complex with larger molecular assemblies, such as those containing AMPARs.

AMPAR containing REs in dendritic spine are important for receptor trafficking and synaptic plasticity (Ehlers, 2000; Park et al., 2004, 2006; Kennedy et al., 2010; Esteves da Silva et al., 2015). AKAP150 palmitoylation is known to regulate AMPAR containing RE trafficking and membrane targeting in dendritic spines (Keith et al., 2012; Woolfrey et al., 2015). While palmitoylated AKAP150 tends to associate with REs in WT synapses (Keith et al., 2012), the loss AKAP150 palmitoylation in AKAP150 CS reduced its colocalization with REs when previously imaged using conventional fluorescence microscopy (Purkey et al., 2018). Here with thick section STEM tomography, we identified AKAP150 immunogold labeled endosomal structures in mouse hippocampal neuron dendritic spines. These AKAP150 labeled endosomal structures in forms of small and large vesicles, coated vesicles, tubules, amorphous membrane compartment, and MVB tubule complexes are in accordance with endosomal structures characterized in dendritic spines of rat CA1 hippocampal pyramidal neurons (Cooney et al., 2002), including large vesicles and tubules endosomes characteristic of REs (Ullrich et al., 1996). In WT spine synapses, AKAP150 labeled endosomal structures counted for vast majority (∼ 86%) of all internal membrane structures in spines; but in CS spine synapses, AKAP150 labeled endosomal structures only counted for ∼45% of all internal membrane structures in spines. We interpret this change as a significant reduction in the association of the palmitoylation-deficient AKAP150 CS mutant with endosomal structures, consistent with previous work (Keith et al., 2012; Woolfrey et al., 2015; Purkey et al., 2018). Since the palmitoyl acyltransferase DHHC2 that modifies AKAP79/150 is localized in REs (Woolfrey et al., 2015), it is possible even non-palmitoylated AKAP would have to at least weakly associate with REs initially to become palmitoylated, but once palmitoylated, the AKAP would stably associate with REs while non-palmitoylated AKAP would dissociate. This might explain the retention of at least some non-palmitoylated AKAP150 in association with REs in the CS mutant, with EM images perhaps revealing snapshots of complex dynamic processes of AKAP150 association with REs. Regardless, our thick section STEM tomography on AKAP150-labeled REs opens new possibilities for future investigation of the processes and mechanisms involving AKAP150 in regulating AMPAR trafficking and synaptic plasticity in dendritic spines.

Hidden-Markov analysis of smtPALM data indicated that the total AKAP79/150 population within the postsynaptic spine is divided between multiple mobility states. Our *d*STORM and EM imaging showed that the distribution of AKAP79/150 molecules is highly heterogenous, with multiple HDRs/nanoclusters arrayed around the periphery of the PSD. While there is still no direct evidence of a link between this spatial heterogeneity and a range of diffusive states, it is reasonable to speculate that AKAP79/150 proteins residing in distinct sub-synaptic compartments and/or associating with different binding partners might exhibit different degrees of mobility. Quite interestingly, at the level of individual AKAP molecules there was an exchange between states (at scales in the 100 s of millisecond range). It is here that the greatest distinction between AKAP79 WT and CS was observed. Evaluation of the relative dwell-times of the apparent “bound” fraction and the transition rates between that state and the slower of two diffusive states suggest that AKAP CS can sample the bound state, but cannot be stabilized to the same degree as WT. AKAP79/150 has multiple synaptic regulatory targets and binding partners (Wild and Dell’Acqua, 2018; Purkey and Dell’Acqua, 2020); of great interest will be determining how these mobility states might correspond to distinct, physical sub-synaptic compartments and molecular complexes within the PSD and/or peri-synaptic/extrasynaptic membrane. The association domains for a number of these interactions, such as with PKA, PKC, CaN, and PSD-95, have been structurally mapped, thus future work could evaluate how disruption of these binding sites may affect the mobility and spatial distribution of AKAP79/150.

AKAP150 is known to be in complex with many ion channels in neurons, such as K_V_, Ca_V_, and TRPV channels (Zhang et al., 2016). In the PSD of excitatory synapses, PSD-95 MAGUKs are regarded as essential players in anchoring glutamate receptors (Chen et al., 2015; Levy et al., 2015), with the complex formed by AKAP150 and PSD-95 (Figure 8) readily allowing access of associated signaling molecules to glutamate receptors at the PSD to modulate LTP or LTD. Accordingly, there are multiple pools of AMPARs in spines, some forming high density clusters at the extrasynaptic membrane (Tao-Cheng et al., 2011) in parallel with the extensive distribution of AKAP79/150 in the same location found in this study. Such an arrangement could allow AKAP79/150 to prominently modulate AMPARs extrasynaptically (Figure 8), thus providing an attractive model for future investigation.

## Materials and methods

This work is approved by the University of Colorado (Denver), and the National Institute of Neurological Disorders and Stroke (NINDS)/The National Institute on Deafness and Other Communication Disorders (NIDCD/The National Center for Complementary and Integrative Health (NCCIH) Animal Care and Use Committees (ACUC).

### Dissociated mouse hippocampal neuron culture

The hippocampi of both male and female AKAP150 WT or CS mice (postnatal day 1–3) were dissected and dissociated in papain. Neurons were plated at a density of 400,000–500,000 cells/well in 6 well dishes on 25 mm glass coverslips coated with poly-D-lysine and laminin (BD Biosciences, San Jose, CA, United States). Cells were maintained at 37°C, 5% CO_2_ in Neurobasal-A medium supplemented with B27, Glutamax, and Pen/Strep for 14–16 days before processing (Purkey et al., 2018).

### Dissociated rat hippocampal neuron culture

The 25 mm round German glass coverslips (Warner Instruments, Holliston, MA, United States) were cleaned with fuming nitric acid and coated with poly-L-lysine (Sigma, St. Louis, MO, United States) before plating. Dissociated rat hippocampal neurons from both male and female E20 rat embryos were layered onto confluent glial feeding layer on glass coverslips (Mayer et al., 1989). Neuronal cultures were fed three times weekly with half changes of MEM (containing Earle’s salts, 6 g/l glucose, 3.7 g/l sodium bicarbonate) supplemented with 5% (v/v) heat-inactivated horse serum, 2% (v/v) fetal bovine serum, 2 mM Glutamax (all from Life Technologies, Carlsbad, CA, United States), 136 mM Uridine 54 mM 2-deoxy-5-fluoro-uridine (Sigma, St. Louis, MO, United States), and N3 supplement containing BSA, apotransferrin, putrescine, selenium, T3, insulin, progesterone, corticosterone (Sigma, St. Louis, MO, United States) (Ransom et al., 1977) and maintained in incubator at 35°C with 10% CO_2_.

### Transfection of GFP tagged AKAP79 constructs in cultured rat hippocampal neurons

21 DIV cultured rat hippocampal neurons were transfected with the AKAP79 constructs using Clontech CalPhos Mammalian Transfection Kit (Chen et al., 2011), typically 2–5 μg of cDNA per 25 mm coverslip was used for transfection and followed by ∼20 h of incubation for protein expression.

### Sample preparation for immunogold electron microscopy

Dissociated mouse or rat hippocampal cultures were fixed in 4% paraformaldehyde in 0.1 M phosphate buffer at pH 7.4 for 45 min at room temperature, then were washed with PBS buffer, and blocked and permeabilized in PBS with 0.1% saponin and 5% normal goat serum for 1 h. Cells were then incubated with either rabbit anti-AKAP150 (Brandao et al., 2012; 1:500) or mouse anti- GFP antibody (Invitrogen 3E6; 1:500) for 1 h and washed, then incubated with secondary antibodies conjugated to 1.4 nm gold (Nanogold, Nanoprobes, Yaphank, NY, United States) for 1 h, washed, and fixed with 2% glutaraldehyde in PBS. Nanogold (1.4 nm) was silver enhanced for 6 or 8 min (HQ silver enhancement kit, Nanoprobes, Yaphank, NY, United States), then sample was treated with 0.2% osmium tetroxide in 0.1 M phosphate buffer on ice for 30 min and then with 0.25% uranyl acetate overnight, washed, dehydrated in series of dilution of ethanol, and embedded in Epon (details see Chen et al., 2018). The glass coverslip was released from the Epon block by brief immersion in liquid nitrogen and the marked area was cut out with a saw and mounted for thin sectioning.

### Conventional electron microscopy image acquisition and analysis

For conventional EM morphological analysis, the section was cut *en face* to a thickness of ∼70 nm and grid stained with UA and lead citrate. No specific labeling was detected when primary antibody was omitted from the protocol. Thin-section EM grids loaded in a JEOL 200CX (operated at 120 kV) or 1,400 transmission electron microscope (operated at 80 kV) were imaged with a bottom-mounted advanced microscopy technique (AMT, Woburn, MA, United States) CCD camera. All distance measurements from electron micrographs were done offline and are reported as means ± SD unless otherwise indicated. Statistical analysis and visualization of all measurements were performed with Prism (GraphPad, San Diego, CA, United States).

### Thick section scanning transmission electron microscopy tomography

The sections were cut to a thickness of 1–1.5 um thick *enface* and mounted on Pioloform F coated 200 mesh hexagonal grids unstained for STEM tomography, carbon-coated and were exposed on both sides to antibody-conjugated 20-nm colloidal gold particles as fiducial markers (in some instances the fiducial gold particles were omitted due to the existence of immunogold in the plastic sections). Thick-section STEM tomography was performed using an FEI Tecnai FT30 300-kV transmission electron microscope equipped with a field emission gun and axial bright field STEM detector (Gatan, Pleasanton, CA, United States). The sections were irradiated with a broad TEM beam for ∼60 min before data acquisition in the bright field STEM mode. The electron dose was ∼10^4^ electrons/nm^2^ per image at the magnifications of either 2.89 nm/pixel or 4.13 nm/pixel (2,048 by 2,048-pixel image). The sample was typically tilted from -60° to + 60° in 2° increments. After the first series was taken, the grid was rotated 90° for the second series. Dual-axis tomography series were individually reconstructed in IMOD (Kremer et al., 1996) (University of Colorado, Boulder, CO) using the weighted back projection algorithm and were combined to generate the final dual-axis tomograms.

### Analysis of thick section scanning transmission electron microscopy tomograms

Tomograms were analyzed with EM3D (Harlow et al., 2001) and IMOD with slicer and segmented and surface rendered with Amira (Thermo Fisher Scientific). Averaged projection images used for membrane-to-immunogold distance measurements, immunogold gold cluster analysis, nearest neighbor distance measurements were generated in EM3D of averaged consecutive virtual slices from thick section tomograms. The number of consecutive virtual sections used for averaging: 3–5 (distance to membrane measurements; endosomes); 50 (nearest neighbor analysis); 50 gold cluster analysis. Segmentation of tomograms, which are stacks of 2D slices of 3D reconstructions of structure of interest were manually traced using Amira (Chen et al., 2008a,b, 2014). Every structure element was segmented in its three orthogonal views in the tomogram all measurements are reported as means ± SD unless otherwise indicated. Statistical analysis and visualization of all measurements were performed with Prism (GraphPad, San Diego, CA, United States).

### Single-molecule fluorescence imaging

#### dSTORM

Primary mouse hippocampal culturing and fixation of wild-type and AKAP150 CS mice is described above and in detail in Purkey et al. (2018). Neurons were labeled with primary antibodies: rabbit anti-AKAP150 (Brandao et al., 2012; 1:1,000) and mouse anti-PSD95 (Millipore; 1:500). After incubation at room temperature, samples were incubated with secondary antibodies: goat anti-rabbit-CF568, 1:500 and goat anti-mouse-Alexa 647, 1:500 (Rockland, Pottstown, PA, United States).

*d*STORM imaging, processing, and analysis has been described in detail (Gookin et al., 2022). Briefly, samples were imaged in a standard *d*STORM buffer and imaging was performed on a Zeiss Elyra P.1 TIRF microscope using a Zeiss alpha Plan Apochromat TIRF 63x/1.46 NA oil objective and a tube lens providing an extra factor of 1.6x magnification. Alexa647 and CF568 dyes were imaged in two sequential time-series of approximately 10,000 frames (50 ms integration time) each. Image size was 256 × 256 pixels (pixel size 160 nm). Dark-state conversion and imaging was done using a 100 mW 642 laser (LP 655 emission filter) and a 200 mW 561 laser (BP 570-650 + LP 750 filter). An additional MBS (405/488/561/642) filter was placed in from of the camera, an Andor iXon + 897 EMCCD.

Raw data was processed through a custom written pipeline written in MATLAB (Mathworks, Natick, MA, United States) made up of a number of modular elements: if necessary, the time-series was pre-processed with a temporal filter (Hoogendoorn et al., 2014). Localization of dye emitters was performed using the ThunderSTORM ImageJ plugin (Ovesny et al., 2014). Registration and drift-correction was based on the positions of fiducial beads (Tetraspeck, Thermo Fisher Scientific).

Our coordinate analysis of *d*STORM localizations is conceptually similar to methods previously used to classify nanoscale organization at excitatory synapses (Tang et al., 2016), using a local-density calculation, high-density regions (HDRs) were defined by a cutoff determined by randomizing the experimental localizations assuming a uniform distribution across the synaptic region, set at 2.5 standard deviations above the mean of the randomized dataset.

#### Single-molecule tracking photo-activation localization microscopy

Primary rat hippocampal neurons were transfected with constructs encoding either AKAP79 WT or AKAP79 CS fused to mEos3.2 (Zhang et al., 2012) along with an AKAP150 RNAi construct to knockdown expression of endogenous rat AKAP150 (Hoshi et al., 2005) at DIV 14–15. Two days later, imaging was performed on the same Elyra P.1 TIRF instrument as our dSTORM experiments. Photoconversion of mEos3.2 was driven by a 405 laser at 0.001–0.1% AOTF transmission; imaging of photoconverted (red-state) mEos3.2 was done with the 200 mW 561 laser at 30–40% AOTF transmission (∼0.2–0.4 W/cm^2^). 10,000 frames were acquired at ∼50 Hz, 20 ms integration time for a total time-step of 22 ms. Localization and linkage steps were performed using the method/software described in Schwartz et al. (2017). Hidden-Markov analysis of pooled trajectories was done using the previously described method (Persson et al., 2013). Only trajectories from distinct spine heads were used in the analysis. For analysis of WT AKAP79, 7303 trajectories (total 1dT jumps: 39,423) from 98 spine ROIs (33 cells from 6 culture sets); for analysis of WT AKAP79, 4,527 trajectories (total 1dT jumps: 24,320) from 50 spine ROIs (14 cells from 3 culture sets).

## Data availability statement

The original contributions presented in this study are included in the article/supplementary material, further inquiries can be directed to the corresponding authors.

## Ethics statement

This animal study was reviewed and approved by the University of Colorado (Denver), and the National Institute of Neurological Disorders and Stroke (NINDS)/The National Institute on Deafness and Other Communication Disorders [NIDCD/The National Center for Complementary and Integrative Health (NCCIH) Animal Care and Use Committees (ACUC)].

## Author contributions

XC, KC, and MD’A designed the experiments. XC, KC, AF, AP, MA, CW, and VC conducted research. RL contributed to the electron microscopy. XC, KC, and AF performed data analysis. XC, KC, AF, and MD’A wrote initial sections of manuscript. All authors contributed to the manuscript revision and approved the final version.

## References

[B1] Bhattacharyya S. Biou V. Xu W. Schlüter O. Malenka R. C. (2009). A critical role for PSD-95/AKAP interactions in endocytosis of synaptic AMPA receptors. *Nat. Neurosci.* 12 172–181. 10.1038/nn.2249 19169250 PMC2694745

[B2] Brandao K. E. Dell’Acqua M. L. Levinson S. R. (2012). A-kinase anchoring protein 150 expression in a specific subset of TRPV1- and CaV1.2-positive nociceptive rat dorsal root ganglion neurons. *J. Comp. Neurol.* 520 81–99. 10.1002/cne.22692 21674494 PMC4807902

[B3] Broadhead M. J. Horrocks M. H. Zhu F. Muresan L. Benavides-Piccione R. DeFelipe J. (2016). PSD95 nanoclusters are postsynaptic building blocks in hippocampus circuits. *Sci. Rep.* 6:24626. 10.1038/srep24626 27109929 PMC4842999

[B4] Chen X. Levy J. M. Hou A. Winters C. Azzam R. Sousa A. A. (2015). PSD-95 family MAGUKs are essential for anchoring AMPA and NMDA receptor complexes at the postsynaptic density. *Proc. Natl. Acad. Sci. U.S.A.* 112 E6983–E6992. 10.1073/pnas.1517045112 26604311 PMC4687590

[B5] Chen X. Nelson C. D. Li X. Winters C. A. Azzam R. Sousa A. A. (2011). PSD-95 is required to sustain the molecular organization of the postsynaptic density. *J. Neurosci.* 31 6329–6338. 10.1523/JNEUROSCI.5968-10.2011 21525273 PMC3099547

[B6] Chen X. Vinade L. Leapman R. D. Petersen J. D. Nakagawa T. Phillips T. M. (2005). Mass of the postsynaptic density and enumeration of three key molecules. *Proc. Natl. Acad. Sci. U.S.A.* 102 11551–11556. 10.1073/pnas.0505359102 16061821 PMC1182136

[B7] Chen X. Winters C. Azzam R. Li X. Galbraith J. A. Leapman R. D. (2008a). Organization of the core structure of the postsynaptic density. *Proc. Natl. Acad. Sci. U.S.A.* 105 4453–4458. 18326622 10.1073/pnas.0800897105 PMC2393784

[B8] Chen X. Winters C. A. Reese T. S. (2008b). Life inside a thin section: Tomography. *J. Neurosci.* 28 9321–9327. 10.1523/JNEUROSCI.2992-08.2008 18799665 PMC2716091

[B9] Chen X. Winters C. Crocker V. Lazarou M. Sousa A. A. Leapman R. D. (2018). Identification of PSD-95 in the postsynaptic density using MiniSOG and EM tomography. *Front. Neuroanat.* 12:107. 10.3389/fnana.2018.00107 30581381 PMC6292990

[B10] Chen X. B. Winters C. Azzam R. Sousa A. A. Leapman R. D. Reese T. S. (2014). Nanoscale imaging of protein molecules at the postsynaptic density. *Nanoscale Imaging Synapses* 84 1–21. 10.1007/978-1-4614-9179-8_1

[B11] Cheng D. Hoogenraad C. C. Rush J. Ramm E. Schlager M. A. Duong D. M. (2006). Relative and absolute quantification of postsynaptic density proteome isolated from rat forebrain and cerebellum. *Mol. Cell. Proteomics* 5 1158–1170. 10.1074/mcp.D500009-MCP200 16507876

[B12] Colledge M. Dean R. A. Scott G. K. Langeberg L. K. Huganir R. L. Scott J. D. (2000). Targeting of PKA to glutamate receptors through a MAGUK-AKAP complex. *Neuron* 27 107–119. 10.1016/S0896-6273(00)00013-1 10939335

[B13] Cooney J. R. Hurlburt J. L. Selig D. K. Harris K. M. Fiala J. C. (2002). Endosomal compartments serve multiple hippocampal dendritic spines from a widespread rather than a local store of recycling membrane. *J. Neurosci.* 22 2215–2224. 10.1523/JNEUROSCI.22-06-02215.2002 11896161 PMC6758269

[B14] Delint-Ramirez I. Willoughby D. Hammond G. V. Ayling L. J. Cooper D. M. (2011). Palmitoylation targets AKAP79 protein to lipid rafts and promotes its regulation of calcium-sensitive adenylyl cyclase type 8. *J. Biol. Chem.* 286 32962–32975. 10.1074/jbc.M111.243899 21771783 PMC3190942

[B15] Dell’Acqua M. L. Faux M. C. Thorburn J. Thorburn A. Scott J. D. (1998). Membrane-targeting sequences on AKAP79 bind phosphatidylinositol-4,5-bisphosphate. *EMBO J.* 17 2246–2260. 10.1093/emboj/17.8.2246 9545238 PMC1170569

[B16] Ehlers M. D. (2000). Reinsertion or degradation of AMPA receptors determined by activity-dependent endocytic sorting. *Neuron* 28 511–525. 11144360 10.1016/s0896-6273(00)00129-x

[B17] Esteves da Silva M. Adrian M. Schatzle P. Lipka J. Watanabe T. Cho S. (2015). Positioning of AMPA receptor-containing endosomes regulates synapse architecture. *Cell Rep.* 13 933–943. 10.1016/j.celrep.2015.09.062 26565907

[B18] Fukata Y. Dimitrov A. Boncompain G. Vielemeyer O. Perez F. Fukata M. (2013). Local palmitoylation cycles define activity-regulated postsynaptic subdomains. *J. Cell Biol.* 202 145–161. 10.1083/jcb.201302071 23836932 PMC3704990

[B19] Gold M. G. Stengel F. Nygren P. J. Weisbrod C. R. Bruce J. E. Robinson C. V. (2011). Architecture and dynamics of an A-kinase anchoring protein 79 (AKAP79) signaling complex. *Proc. Natl. Acad. Sci. U.S.A.* 108 6426–6431. 10.1073/pnas.1014400108 21464287 PMC3081024

[B20] Gomez L. L. Alam S. Smith K. E. Horne E. Dell’Acqua M. L. (2002). Regulation of A-Kinase anchoring protein 79/150–cAMP-dependent protein kinase postsynaptic targeting by NMDA receptor activation of calcineurin and remodeling of dendritic actin. *J. Neurosci.* 22 7027–7044. 10.1523/jneurosci.22-16-07027.2002 12177200 PMC6757891

[B21] Gookin S. E. Taylor M. R. Schwartz S. L. Kennedy M. J. Dell’Acqua M. L. Crosby K. C. (2022). Complementary use of super-resolution imaging modalities to study the nanoscale architecture of inhibitory synapses. *Front. Synaptic Neurosci.* 14:852227. 10.3389/fnsyn.2022.852227 35463850 PMC9024107

[B22] Gorski J. A. Gomez L. L. Scott J. D. Dell’Acqua M. L. (2005). Association of an A-kinase-anchoring protein signaling scaffold with cadherin adhesion molecules in neurons and epithelial cells. *Mol. Biol. Cell* 16 3574–3590. 10.1091/mbc.e05-02-0134 15930126 PMC1182299

[B23] Hansen A. S. Woringer M. Grimm J. B. Lavis L. D. Tjian R. Darzacq X. (2018). Robust model-based analysis of single-particle tracking experiments with Spot-On. *eLife* 7:e33125. 10.7554/eLife.33125 29300163 PMC5809147

[B24] Harlow M. L. Ress D. Stoschek A. Marshall R. M. McMahan U. J. (2001). The architecture of active zone material at the frog’s neuromuscular junction. *Nature* 409 479–484. 10.1038/35054000 11206537

[B25] Harris K. M. Jensen F. E. Tsao B. (1992). Three-dimensional structure of dendritic spines and synapses in rat hippocampus (CA1) at postnatal day 15 and adult ages: Implications for the maturation of synaptic physiology and long-term potentiation. *J. Neurosci.* 12 2685–2705. 10.1523/JNEUROSCI.12-07-02685.1992 1613552 PMC6575840

[B26] Harris K. M. Stevens J. K. (1989). Dendritic spines of CA 1 pyramidal cells in the rat hippocampus: Serial electron microscopy with reference to their biophysical characteristics. *J. Neurosci.* 9 2982–2997. 10.1523/JNEUROSCI.09-08-02982.1989 2769375 PMC6569708

[B27] Hohmann-Marriott M. F. Sousa A. A. Azari A. A. Glushakova S. Zhang G. Zimmerberg J. (2009). Nanoscale 3D cellular imaging by axial scanning transmission electron tomography. *Nat. Methods* 6 729–731. 10.1038/nmeth.1367 19718033 PMC2755602

[B28] Hoogendoorn E. Crosby K. C. Leyton-Puig D. Breedijk R. M. Jalink K. Gadella T. W. (2014). The fidelity of stochastic single-molecule super-resolution reconstructions critically depends upon robust background estimation. *Sci. Rep.* 4:3854. 10.1038/srep03854 24458236 PMC3900998

[B29] Horne E. A. Dell’Acqua M. L. (2007). Phospholipase C is required for changes in postsynaptic structure and function associated with NMDA receptor-dependent long-term depression. *J. Neurosci.* 27 3523–3534. 10.1523/jneurosci.4340-06.2007 17392468 PMC6672111

[B30] Hoshi N. Langeberg L. K. Scott J. D. (2005). Distinct enzyme combinations in AKAP signalling complexes permit functional diversity. *Nat. Cell Biol.* 7 1066–1073. 10.1038/ncb1315 16228013 PMC3923410

[B31] Hruska M. Henderson N. Le Marchand S. J. Jafri H. Dalva M. B. (2018). Synaptic nanomodules underlie the organization and plasticity of spine synapses. *Nat. Neurosci.* 21 671–682. 10.1038/s41593-018-0138-9 29686261 PMC5920789

[B32] Keith D. J. Sanderson J. L. Gibson E. S. Woolfrey K. M. Robertson H. R. Olszewski K. (2012). Palmitoylation of A-Kinase anchoring protein 79/150 regulates dendritic endosomal targeting and synaptic plasticity mechanisms. *J. Neurosci.* 32 7119–7136. 10.1523/jneurosci.0784-12.2012 22623657 PMC3367663

[B33] Kennedy M. J. Davison I. G. Robinson C. G. Ehlers M. D. (2010). Syntaxin-4 defines a domain for activity-dependent exocytosis in dendritic spines. *Cell* 141 524–535. 10.1016/j.cell.2010.02.042 20434989 PMC2874581

[B34] Kennedy M. J. Ehlers M. D. (2011). Mechanisms and function of dendritic exocytosis. *Neuron* 69 856–875. 10.1016/j.neuron.2011.02.032 21382547 PMC3073864

[B35] Kremer J. R. Mastronarde D. N. McIntosh J. R. (1996). Computer visualization of three-dimensional image data using IMOD. *J. Struct. Biol.* 116 71–76. 10.1006/jsbi.1996.0013 8742726

[B36] Levy J. M. Chen X. Reese T. S. Nicoll R. A. (2015). Synaptic consolidation normalizes AMPAR quantal size following MAGUK loss. *Neuron* 87 534–548. 10.1016/j.neuron.2015.07.015 26247861 PMC4596923

[B37] Lilly S. M. Alvarez F. J. Tietz E. I. (2005). Synaptic and subcellular localization of A-kinase anchoring protein 150 in rat hippocampal CA1 pyramidal cells: Co-localization with excitatory synaptic markers. *Neuroscience* 134 155–163. 10.1016/j.neuroscience.2005.03.039 15951119

[B38] Lu Y. Allen M. Halt A. R. Weisenhaus M. Dallapiazza R. F. Hall D. D. (2007). Age-dependent requirement of AKAP150-anchored PKA and GluR2-lacking AMPA receptors in LTP. *EMBO J.* 26 4879–4890. 10.1038/sj.emboj.7601884 17972919 PMC2099463

[B39] Lu Y. Zhang M. Lim I. A. Hall D. D. Allen M. Medvedeva Y. (2008). AKAP150-anchored PKA activity is important for LTD during its induction phase. *J. Physiol.* 586(Pt 17), 4155–4164. 10.1113/jphysiol.2008.151662 18617570 PMC2652176

[B40] MacGillavry H. D. Song Y. Raghavachari S. Blanpied T. A. (2013). Nanoscale scaffolding domains within the postsynaptic density concentrate synaptic AMPA receptors. *Neuron* 78 615–622. 10.1016/j.neuron.2013.03.009 23719161 PMC3668352

[B41] Manley S. Gillette J. M. Patterson G. H. Shroff H. Hess H. F. Betzig E. (2008). High-density mapping of single-molecule trajectories with photoactivated localization microscopy. *Nat. Methods* 5 155–157. 10.1038/nmeth.1176 18193054

[B42] Martinez-Sanchez A. Laugks U. Kochovski Z. Papantoniou C. Zinzula L. Baumeister W. (2021). Trans-synaptic assemblies link synaptic vesicles and neuroreceptors. *Sci. Adv.* 7:eabe6204. 10.1126/sciadv.abe6204 33674312 PMC7935360

[B43] Masch J. M. Steffens H. Fischer J. Engelhardt J. Hubrich J. Keller-Findeisen J. (2018). Robust nanoscopy of a synaptic protein in living mice by organic-fluorophore labeling. *Proc. Natl. Acad. Sci. U.S.A.* 115 E8047–E8056. 10.1073/pnas.1807104115 30082388 PMC6112726

[B44] Mayer M. L. Vyklicky L. Jr. Westbrook G. L. (1989). Modulation of excitatory amino acid receptors by group IIB metal cations in cultured mouse hippocampal neurones. *J. Physiol.* 415 329–350. 10.1113/jphysiol.1989.sp017724 2561788 PMC1189179

[B45] Michalet X. Berglund A. J. (2012). Optimal diffusion coefficient estimation in single-particle tracking. *Phys. Rev. E Stat. Nonlin. Soft Matter Phys.* 85:061916. 10.1103/PhysRevE.85.061916 23005136 PMC4917385

[B46] Nair D. Hosy E. Petersen J. D. Constals A. Giannone G. Choquet D. (2013). Super-resolution imaging reveals that AMPA receptors inside synapses are dynamically organized in nanodomains regulated by PSD95. *J. Neurosci.* 33 13204–13224. 10.1523/jneurosci.2381-12.2013 23926273 PMC6619720

[B47] Nicoll R. A. (2017). A brief history of long-term potentiation. *Neuron* 93 281–290. 10.1016/j.neuron.2016.12.015 28103477

[B48] Nygren P. J. Mehta S. Schweppe D. K. Langeberg L. K. Whiting J. L. Weisbrod C. R. (2017). Intrinsic disorder within AKAP79 fine-tunes anchored phosphatase activity toward substrates and drug sensitivity. *eLife* 6:e30872. 10.7554/eLife.30872 28967377 PMC5653234

[B49] Opazo P. Sainlos M. Choquet D. (2012). Regulation of AMPA receptor surface diffusion by PSD-95 slots. *Curr. Opin. Neurobiol.* 22 453–460. 10.1016/j.conb.2011.10.010 22051694

[B50] Ovesny M. Krizek P. Borkovec J. Svindrych Z. Hagen G. M. (2014). ThunderSTORM: A comprehensive ImageJ plug-in for PALM and STORM data analysis and super-resolution imaging. *Bioinformatics* 30 2389–2390. 10.1093/bioinformatics/btu202 24771516 PMC4207427

[B51] Park M. Penick E. C. Edwards J. G. Kauer J. A. Ehlers M. D. (2004). Recycling endosomes supply AMPA receptors for LTP. *Science* 305 1972–1975. 15448273 10.1126/science.1102026

[B52] Park M. Salgado J. M. Ostroff L. Helton T. D. Robinson C. G. Harris K. M. (2006). Plasticity-induced growth of dendritic spines by exocytic trafficking from recycling endosomes. *Neuron* 52 817–830. 10.1016/j.neuron.2006.09.040 17145503 PMC1899130

[B53] Penn A. C. Zhang C. L. Georges F. Royer L. Breillat C. Hosy E. (2017). Hippocampal LTP and contextual learning require surface diffusion of AMPA receptors. *Nature* 549 384–388. 10.1038/nature23658 28902836 PMC5683353

[B54] Persson F. Lindén M. Unoson C. Elf J. (2013). Extracting intracellular diffusive states and transition rates from single-molecule tracking data. *Nat. Methods* 10 265–269. 10.1038/nmeth.2367 23396281

[B55] Petralia R. S. Wenthold R. J. (1999). Immunocytochemistry of NMDA receptors. *Methods Mol. Biol.* 128 73–92. 10320974 10.1385/1-59259-683-5:73

[B56] Purkey A. M. Dell’Acqua M. L. (2020). Phosphorylation-dependent regulation of Ca2+-permeable AMPA receptors during hippocampal synaptic plasticity. *Front. Synaptic Neurosci.* 12:8. 10.3389/fnsyn.2020.00008 32292336 PMC7119613

[B57] Purkey A. M. Woolfrey K. M. Crosby K. C. Stich D. G. Chick W. S. Aoto J. (2018). AKAP150 palmitoylation regulates synaptic incorporation of Ca^2+^-permeable AMPA receptors to control LTP. *Cell Rep.* 25 974–987.e4. 10.1016/j.celrep.2018.09.085 30355502 PMC6263960

[B58] Ransom B. R. Neale E. Henkart M. Bullock P. N. Nelson P. G. (1977). Mouse spinal cord in cell culture. I. Morphology and intrinsic neuronal electrophysiologic properties. *J. Neurophysiol.* 40 1132–1150. 10.1152/jn.1977.40.5.1132 333062

[B59] Robertson H. R. Gibson E. S. Benke T. A. Dell’Acqua M. L. (2009). Regulation of postsynaptic structure and function by an A-kinase anchoring protein–membrane-associated guanylate kinase scaffolding complex. *J. Neurosci.* 29 7929–7943. 10.1523/jneurosci.6093-08.2009 19535604 PMC2716089

[B60] Sanderson J. L. Freund R. K. Gorski J. A. Dell’Acqua M. L. (2021). β-Amyloid disruption of LTP/LTD balance is mediated by AKAP150-anchored PKA and Calcineurin regulation of Ca2+-permeable AMPA receptors. *Cell Rep.* 37:109786. 10.1016/j.celrep.2021.109786 34610314 PMC8530450

[B61] Sanderson J. L. Gorski J. A. Dell’Acqua M. L. (2016). NMDA receptor-dependent LTD requires transient synaptic incorporation of Ca2+-permeable AMPARs mediated by AKAP150-anchored PKA and calcineurin. *Neuron* 89 1000–1015. 10.1016/j.neuron.2016.01.043 26938443 PMC4914360

[B62] Sanderson J. L. Gorski J. A. Gibson E. S. Lam P. Freund R. K. Chick W. S. (2012). AKAP150-anchored calcineurin regulates synaptic plasticity by limiting synaptic incorporation of Ca^2+^-permeable AMPA receptors. *J. Neurosci.* 32 15036–15052. 10.1523/jneurosci.3326-12.2012 23100425 PMC3504485

[B63] Sanderson J. L. Scott J. D. Dell’Acqua M. L. (2018). Control of homeostatic synaptic plasticity by AKAP-anchored kinase and phosphatase regulation of Ca^2+^-permeable AMPA receptors. *J. Neurosci.* 38 2863–2876. 10.1523/jneurosci.2362-17.2018 29440558 PMC5852664

[B64] Schwartz S. L. Cleyrat C. Olah M. J. Relich P. K. Phillips G. K. Hlavacek W. S. (2017). Differential mast cell outcomes are sensitive to FcεRI-Syk binding kinetics. *Mol. Biol. Cell* 28 3397–3414. 10.1091/mbc.e17-06-0350 28855374 PMC5687039

[B65] Sik A. Gulacsi A. Lai Y. Doyle W. K. Pacia S. Mody I. (2000). Localization of the A kinase anchoring protein AKAP79 in the human hippocampus. *Eur. J. Neurosci.* 12 1155–1164. 10.1046/j.1460-9568.2000.00002.x 10762347

[B66] Tang A.-H. Chen H. Li T. P. Metzbower S. R. MacGillavry H. D. Blanpied T. A. (2016). A trans-synaptic nanocolumn aligns neurotransmitter release to receptors. *Nature* 536 210–214. 10.1038/nature19058 27462810 PMC5002394

[B67] Tao-Cheng J. H. Crocker V. Moreira S. L. Azzam R. (2021). Optimization of protocols for pre-embedding immunogold electron microscopy of neurons in cell cultures and brains. *Mol. Brain* 14:86. 10.1186/s13041-021-00799-2 34082785 PMC8173732

[B68] Tao-Cheng J. H. Crocker V. T. Winters C. A. Azzam R. Chludzinski J. Reese T. S. (2011). Trafficking of AMPA receptors at plasma membranes of hippocampal neurons. *J. Neurosci.* 31 4834–4843. 10.1523/JNEUROSCI.4745-10.2011 21451021 PMC3138201

[B69] Tenner B. Getz M. Ross B. Ohadi D. Bohrer C. H. Greenwald E. (2020). Spatially compartmentalized phase regulation of a Ca(2+)-cAMP-PKA oscillatory circuit. *eLife* 9:e55013. 10.7554/eLife.55013 33201801 PMC7671691

[B70] Ullrich O. Reinsch S. Urbe S. Zerial M. Parton R. G. (1996). Rab11 regulates recycling through the pericentriolar recycling endosome. *J. Cell Biol.* 135 913–924. 10.1083/jcb.135.4.913 8922376 PMC2133374

[B71] Valtschanoff J. G. Weinberg R. J. (2001). Laminar organization of the NMDA receptor complex within the postsynaptic density. *J. Neurosci.* 21 1211–1217. 11160391 10.1523/JNEUROSCI.21-04-01211.2001 PMC6762240

[B72] Wegner W. Mott A. C. Grant S. G. N. Steffens H. Willig K. I. (2018). In vivo STED microscopy visualizes PSD95 sub-structures and morphological changes over several hours in the mouse visual cortex. *Sci. Rep.* 8:219. 10.1038/s41598-017-18640-z 29317733 PMC5760696

[B73] Wild A. R. Dell’Acqua M. L. (2018). Potential for therapeutic targeting of AKAP signaling complexes in nervous system disorders. *Pharmacol. Ther.* 185 99–121. 10.1016/j.pharmthera.2017.12.004 29262295 PMC5899024

[B74] Wong W. Scott J. D. (2004). AKAP signalling complexes: Focal points in space and time. *Nat. Rev. Mol. Cell Biol.* 5 959–970. 10.1038/nrm1527 15573134

[B75] Woolfrey K. M. O’Leary H. Goodell D. J. Robertson H. R. Horne E. A. Coultrap S. J. (2018). CaMKII regulates the depalmitoylation and synaptic removal of the scaffold protein AKAP79/150 to mediate structural long-term depression. *J. Biol. Chem.* 293 1551–1567. 10.1074/jbc.M117.813808 29196604 PMC5798287

[B76] Woolfrey K. M. Sanderson J. L. Dell’Acqua M. L. (2015). The palmitoyl acyltransferase DHHC2 regulates recycling endosome exocytosis and synaptic potentiation through palmitoylation of AKAP79/150. *J. Neurosci.* 35 442–456. 10.1523/jneurosci.2243-14.2015 25589740 PMC4293401

[B77] Xu W. Schluter O. M. Steiner P. Czervionke B. L. Sabatini B. Malenka R. C. (2008). Molecular dissociation of the role of PSD-95 in regulating synaptic strength and LTD. *Neuron* 57 248–262. 10.1016/j.neuron.2007.11.027 18215622 PMC3147180

[B78] Zhang J. Carver C. M. Choveau F. S. Shapiro M. S. (2016). Clustering and functional coupling of diverse ion channels and signaling proteins revealed by super-resolution STORM microscopy in neurons. *Neuron* 92 461–478. 10.1016/j.neuron.2016.09.014 27693258 PMC5553284

[B79] Zhang M. Chang H. Zhang Y. Yu J. Wu L. Ji W. (2012). Rational design of true monomeric and bright photoactivatable fluorescent proteins. *Nat. Methods* 9 727–729. 10.1038/nmeth.2021 22581370

